# PRAME Updated: Diagnostic, Prognostic, and Therapeutic Role in Skin Cancer

**DOI:** 10.3390/ijms25031582

**Published:** 2024-01-27

**Authors:** Fortunato Cassalia, Andrea Danese, Ina Tudurachi, Serena Federico, Anna Zambello, Alessia Guidotti, Ludovica Franceschin, Anna Bolzon, Luigi Naldi, Anna Belloni Fortina

**Affiliations:** 1 Dermatology Unit, Department of Medicine (DIMED), University of Padua, 35121 Padua, Italy; ina.tudurachi@gmail.com (I.T.); zambello-anna@hotmail.it (A.Z.); alessia.guidotts@gmail.com (A.G.); ludovica.franceschin@gmail.com (L.F.); annabolzon44@gmail.com (A.B.); anna.bellonifortina@gmail.com (A.B.F.); 2 Dermatology Unit, Department of Integrated Medical and General Activity, University of Verona, 37100 Verona, Italy; adanese4@gmail.com; 3 Dermatology Unit, University of Magna Graecia, 88100 Catanzaro, Italy; drserenafederico@gmail.com; 4 Department of Dermatology, Ospedale San Bortolo, 36100 Vicenza, Italy; luiginaldibg@gmail.com; 5 Centro Studi Gruppo Italiano Studi Epidemiologici in Dermatologia (GISED), 24121 Bergamo, Italy; 6 Pediatric Dermatology Department of Women’s and Child’s Health (SDB), University of Padua, 35121 Padua, Italy

**Keywords:** PRAME, melanoma, NMSC, Spitz tumour, uveal melanoma, mucosal melanoma, acral melanoma, soft tissue tumour

## Abstract

Preferentially Expressed Antigen in Melanoma (PRAME), a member of the cancer/testis antigen family, is central to the field of skin cancer diagnostics and therapeutics. As a nuclear receptor and transcriptional regulator, PRAME plays a critical role in inhibiting retinoic acid signalling, which is essential for cell differentiation and proliferation. Its aberrant overexpression in various malignancies, particularly cutaneous melanoma, is associated with more aggressive tumour phenotypes, positioning PRAME as both a diagnostic and prognostic marker. In melanoma, PRAME is typically highly expressed, in contrast to its weak or absent expression in benign nevi, thereby improving the accuracy of differential diagnoses. The diagnostic value of PRAME extends to various lesions. It is significantly expressed in uveal melanoma, correlating to an increased risk of metastasis. In acral melanomas, especially those with histopathological ambiguity, PRAME helps to improve diagnostic accuracy. However, its expression in spitzoid and ungual melanocytic lesions is inconsistent and requires a comprehensive approach for an accurate assessment. In soft tissue sarcomas, PRAME may be particularly helpful in differentiating melanoma from clear cell sarcoma, an important distinction due to their similar histological appearance but different treatment approaches and prognosis, or in detecting dedifferentiated and undifferentiated melanomas. In non-melanoma skin cancers such as basal cell carcinoma, squamous cell carcinoma, and Merkel cell carcinoma, the variable expression of PRAME can lead to diagnostic complexity. Despite these challenges, the potential of PRAME as a therapeutic target in melanoma is significant. Emerging immunotherapies, including T-cell-based therapies and vaccines targeting PRAME, are being investigated to exploit its cancer-specific expression. Ongoing research into the molecular role and mechanism of action of PRAME in skin cancer continues to open new avenues in both diagnostics and therapeutics, with the potential to transform the management of melanoma and related skin cancers.

## 1. Introduction

Preferentially expressed Antigen in Melanoma (PRAME), also known as CT130 (cancer testis antigen 130), MAPE (melanoma antigen preferentially expressed in tumours), and OIP-4 (Opa-interacting protein 4) [1], belongs to the cancer/testis antigen (CTA) gene family [2] and encodes a membrane-bound protein recognised by T lymphocytes, which is a cancer-testis antigen that was first identified in 1997 in cutaneous melanoma by Ikeda et al. [3]. PRAME is a member of a class of tumour-associated antigens. Normal healthy tissues are not known to express PRAME, except for the testis, ovary, placenta, adrenals, and endometrium (Table 1), but it is aberrantly expressed in various malignancies (Table 2) [4,5]. PRAME functions as an inhibitor of retinoic acid signalling, which plays a role in cell differentiation and proliferation [6]. The expression of PRAME is a marker for an undifferentiated cellular state and has been implicated in the inhibition of tumour cell differentiation and the promotion of proliferation, which can contribute to oncogenesis [2].

## 2. Molecular Background

PRAME is a nuclear receptor, a transcriptional regulator, and a member of the cancer testis antigen (CTA) family of proteins. It is situated on the reverse strand of chromosome 22 (22q11.22), within the human immunoglobulin lambda gene locus. It is located amongst various non-immunoglobulin genes, positioned between the tandem Suppressor of Hairy Wing genes (SUHW1/ZNF280A and SUHW2/ZNF280B) and a gene encoding potential. While there are indications that AML1-ETO and BCR-ABL fusion proteins might contribute to PRAME upregulation [9], there are contrasting reports suggesting that the membrane glycoprotein (POM121L1) [10], adjacent to POM121L1, is the pseudogene BCR4 (or BCR4L), recognised as a breakpoint cluster region implicated in chromosome 22 rearrangements [11]. At least 17 different PRAME mRNAs have been described in sequence databases. Each major transcript consists of six exons, four of which contain coding sequences, all encoding an identical polypeptide of 509 amino acids. Differences in the 5′ ends of these transcripts suggest alternative transcription start sites, supported by strong promoter activity in reporter assays around the proximal transcription start site, including exon 1a and the first intron of the PRAME gene (−165 to +365) [9]. Four out of the five validated PRAME transcripts contain unique 5′ untranslated regions (5′ UTRs). Human PRAME 5′ UTRs can form stable secondary structures, likely inhibiting translation initiation under normal conditions. Structurally, PRAME can be defined as a leucine-rich protein, with leucine or isoleucine constituting 21.8% of its residues. Its typical leucine-rich repeats (LRR) motifs, consisting of 20–30 amino acids, entail a beta sheet followed by an alpha-helix, forming a curved solenoid fold with a parallel beta sheet on the concave side and helical elements on the convex side [12]. However, not all LRRs conform strictly to this consensus, with atypical repeats present in certain families, including the PRAME and NALP families. The tertiary structure of the LRR stack in PRAME offers an optimal module for molecular interactions with proteins, nucleic acids, and other ligands. LRR domains play crucial roles in cell immunity, cell adhesion, and signal transduction. Physiologically, PRAME is a repressor of the retinoic acid (RA) receptor pathway via SOX9, a transcription factor with antiproliferative effects, crucial during embryonic development and in adult tissues [13]. SOX9 causes the direct activation of the microphthalmia transcription factor (MITF) promoter, critical for normal melanocyte differentiation and the modulation of melanoma proliferation. It has been demonstrated that SOX9 reduced PRAME expression, reinstating melanoma cell sensitivity to RA [14]. The regulatory mechanisms governing PRAME gene expression remain poorly elucidated, leading to a limited understanding of its molecular basis in malignancies. SOX9 suppresses PRAME expression [15]. It seems that the PRAME gene undergoes hypermethylation in normal tissues, but experiences hypomethylation in malignant cells. Regarding this, some studies have indicated that this induction correlates with the demethylation of specific cytosine/guanine dinucleotide-rich regions in the PRAME promoter [9]. The idea that PRAME is regulated in a way that is proportional to the malignant potential of cell lines led us to identify the main factors responsible for its regulation. It is known that the expression of PRAME is stimulated by the transcription factor MZF1 in cooperation with DNA hypomethylation. In parallel, 5-azaC is known to co-operate with endogenous MZF1 by increasing DNA hypomethylation in the synergistic induction of PRAME. In addition to MZF1, other potential transcription factors including SOX2, SOX3, and ZNF740 have been identified through the analysis of the JASPAR database (http://jaspar.genereg.net (accessed on 1 November 2023) [15]. In addition, it has been reported that CpG-rich regions are completely unmethylated in PRAME-positive cells, e.g., in patients with acute myeloid leukaemia. In this context, the overexpression of PRAME may be a mechanism by which tumour cells can escape tumour suppressor RAR signalling [6], e.g., by interacting with RAR in a ligand-dependent manner and inhibiting RAR-dependent transactivation by interacting with PcG complexes. Given that PRAME is upregulated in various types of neoplasia, it would be interesting to understand how different factors work together to regulate PRAME expression.

## 3. PRAME and Cancer

The expression of PRAME is not universal among all cancers, but shows a broad pattern of upregulation in many of them [16,17,18]. For instance, high levels of PRAME expression have been documented in cutaneous melanoma, which is where the antigen was first discovered [8,19], but it has since been found to be expressed in various malignancies, including leukaemia [7], neuroblastoma [20], non-small-cell lung cancer [21], breast cancer [22], ovarian cancer [23], and various sarcomas [24,25]. PRAME has not been as widely researched in non-melanoma skin cancers (NMSCs) (Table 2). Also, its expression seems to vary quite vastly among the reported studies, with some showing a strong and diffuse PRAME expression in basal cell skin carcinomas and, more infrequently, in Merkel cell carcinomas (MCCs) [13], while others show a negative or low-intensity PRAME expression in a small percentage of NMSCs, except for some MCCs showing high-intensity and diffuse staining [26]. Each of these cancers can exhibit varying levels of PRAME expression, which may correlate with clinical outcomes. For example, in leukaemia, particularly in acute myeloid leukaemia (AML), high PRAME expression has been associated with higher rates of disease relapse and is often used as a marker to monitor the minimal residual disease [27]. In contrast, PRAME expression in breast and ovarian cancers is less well characterised, but preliminary studies suggest that it may also be associated with a worse prognosis [22,23,28]. Comparing PRAME expression across these cancer types reveals the complexity of its role in oncogenesis. In melanoma and AML, the high expression of PRAME is generally indicative of a more aggressive phenotype and is considered an adverse prognostic factor [29]. However, in other cancers, the prognostic significance of PRAME expression is less clear, and further research is needed to understand its role fully. What is intriguing about PRAME is its potential as a therapeutic target. Given its preferential expression in cancer cells, it is an attractive target for immunotherapy approaches, such as cancer vaccines or Chimeric Antigen Receptor T-cell (CAR-T) therapies [30,31]. These strategies aim to train the immune system to recognise and destroy cells expressing PRAME, sparing normal tissues that do not express the antigen or express it at low levels [32,33]. Several clinical trials are underway trying to exploit CTAs, including PRAME, for cancer treatment. Additionally, many studies propose that PRAME expression in tumour cells renders these cells resistant to normal target therapies [34]. These mechanisms should be further studied to identify the precise mechanisms of resistance to PRAME-Targeted therapy. 

## 4. PRAME Expression in Melanocytic Lesions

PRAME has proven to be a valuable tool for the accurate diagnosis of melanocytic tumours, due to its unique expression pattern, and a useful marker in the differential diagnosis between malignant melanoma and other melanocytic lesions [34]. It is known that PRAME expression in benign nevi is absent or weakly stained, whereas melanomas often have a strong PRAME expression [34]. In this regard, several studies have identified PRAME as a marker of primary and metastatic melanomas with high sensitivity (>90%) and specificity in the context of melanocytic lesions [35]. In cases of metastatic melanomas, PRAME expression may also help to distinguish lymph node melanoma metastases from benign melanocytic deposits in the lymph nodes, termed nodal nevi [36]. Hence, when dealing with a metastatic disease, relying solely on PRAME expression for diagnosing melanoma is insufficient, and confirmation through supplementary immunohistochemical or molecular analyses is essential. Nevertheless, the assessment of PRAME in metastatic melanoma goes beyond diagnostic significance, potentially holding prognostic value and serving as a prospective target for immunotherapy [37]. Regarding mucosal melanocytic lesions, which are less common than cutaneous ones, several studies have focused on detecting the presence of PRAME within these lesions to assess their applicability [34]. A study by Ricci et al. found that most benign melanocytic lesions of the mucosa of the head and neck do not show positive immunostaining for PRAME. However, a minority of cases show rare positive cells scattered in the lesion without significant changes in the expression intensity or location [38]. On the other hand, a study by Toyama et al. reported that most mucosal melanomas express PRAME and a high PRAME expression correlates with a poor prognosis. Furthermore, despite the association between increased PRAME expression, the presence of pathogenic NRAS mutations, and a worse prognosis in mucosal melanomas, PRAME expression does not appear to correlate with the presence of NRAS mutations, suggesting that although both factors taken individually may be associated with worse outcomes, they may act independently in the context of mucosal melanomas [39]. Raghavan and colleagues identified that the optimal threshold for differentiating benign from malignant tumours is 60% PRAME-positive cells. This threshold, included in the scoring system devised by the author, has been shown to be effective in distinguishing between mucosal melanomas and benign melanocytic lesions in the head and neck region. In conclusion, PRAME is more commonly expressed in malignant mucosal melanocytic lesions and its increased expression is associated with a poor prognosis. This factor appears to be a prognostic indicator independent of other factors, such as NRAS mutations, and its expression does not appear to correlate with the specific location of a malignant mucosal lesion [34,38,39]. PRAME expression is highly sensitive and specific in the context of acral melanomas and is a more predictive diagnostic tool than p16 immunohistochemistry. Acral dysplastic nevi and acral Spitz nevi may show significant architectural and cellular atypia (a large nuclear size, irregularly shaped nuclei, hyperchromatic nuclei, or prominent nucleoli), which makes them difficult to distinguish from acral melanoma. The study by Jeffrey D. McBride et al. shows that all compounds, dysplastic and Spitz nevi, were negative for PRAME expression. All melanomas showed PRAME 4+ expression. In acral melanomas, PRAME was 4+ positive in a wide range of Breslow thicknesses, from thin melanomas (Breslow 0.4 mm) to thick melanomas (Breslow at least 13 mm) [40]. In thicker melanomas, however, there was regional variation in the intensity of the nuclear expression of PRAME, equalling 4+ PRAME. According to this study, in cases with 1+ to 3+ staining, additional ancillary tests may be important, depending on the associated clinicopathological features. The results give further confidence in the use of PRAME immunohistochemical staining in the evaluation of melanocytic malignancies. According to the literature, especially in acral melanomas characterised by doubtful or misleading histopathological features, PRAME assumes an important role as an immunohistochemical marker that, although not capable of confirming the diagnosis, is able to support the histological diagnosis [41]. PRAME is an intriguing biomarker and a potential therapeutic target in the field of ocular melanomas, particularly uveal and conjunctival melanomas.^4,5^ Understanding its role and implications in these diseases requires a deeper understanding of the molecular biology of melanomas, the specific features of uveal and conjunctival melanomas, and emerging therapeutic strategies targeting PRAME [42,43]. Uveal melanoma originates from melanocytes of the uvea, which includes the iris, ciliary body, and choroid. It differs from cutaneous melanoma in genetics, behaviour, and response to treatment. Unlike cutaneous melanoma, which often involves mutations in the BRAF gene, uveal melanoma commonly has mutations in the GNAQ and GNA11 genes [43]. The overexpression of PRAME in uveal melanoma has been linked to a worse prognosis and an increased risk of metastasis, particularly in the liver [44]. Conjunctival melanoma, although rarer than uveal melanoma, is equally serious and arises from the conjunctiva covering the white part of the eye and inner eyelids. It shares some histopathological features with uveal and cutaneous melanomas [45]. The role of PRAME in conjunctival melanoma is less well known, but is being explored as a potential biomarker for early diagnosis and prognosis [46]. The overexpression of PRAME in melanomas represents a significant opportunity for cancer immunotherapy. Strategies to exploit this target include cancer vaccines, which stimulate the immune system to recognise and attack PRAME-expressing tumour cells, using PRAME peptides to elicit a more targeted and robust anti-tumour response [30]. Furthermore, it is believed that the integration of checkpoint inhibitors with PRAME-targeted therapies could potentially enhance the immune system’s response to the tumour [31]. This synergy could be due to PRAME’s influence on the tumour’s immune environment, suggesting a holistic approach to enhance the body’s natural defence against cancer [32]. The role of PRAME in ocular melanomas and its potential as a therapeutic target represent interesting opportunities. However, challenges remain. The variability of PRAME expression among different tumours, the understanding of its precise role in tumour biology, and the optimisation of immunotherapeutic strategies to target PRAME are areas of active research [47]. Future studies focusing on molecular pathways involving PRAME and clinical trials exploring PRAME-targeted therapies will shed more light on its potential in the management of uveal and conjunctival melanoma. Subungual melanoma can also be difficult to diagnose and PRAME could help in the diagnostic process. Several studies have evaluated the potential of PRAME immunoreactivity to differentiate benign subungual melanocytic proliferation (SMP) from malignant subungual melanocytic lesions, with relative sensitivity and high specificity in differentiating benign from malignant subungual melanocytic lesions [48,49,50]. This antibody has also proved to be diagnostically valuable in detecting melanoma cells in small specimens with minimal disease [51]. However, in some cases, such as acral Spitz nevi, melanomas in situ, and small, thin, invasive melanomas, PRAME did not correlate with morphological features [48,52]. Furthermore, in some subungual melanomas (SUM) and non-subungual acral melanomas (AM), PRAME expression was negative, whereas in some benign nevi, it was positive [53]. There is little conclusive evidence for the use of PRAME in amelanotic melanoma and further studies are needed to understand its potential use.

## 5. PRAME Expression in Spitzoid Lesions

Interestingly, PRAME immunoreactivity has also been observed in Spitz nevi [54], as well as in solar lentigines and benign uninjured skin [34]. Indeed, an intriguing aspect of PRAME expression is its variability within Spitz nevi [34]. In their 2022 study, Koh et al. observed the strong diffuse staining of PRAME in 20% of Spitz nevi, 0% of atypical Spitz tumours, and 82% of spitzoid melanomas. This study shows a significant discrepancy in the expression of PRAME in spitzoid melanomas compared to Spitz nevi/tumours, demonstrating that it may be a valuable additional tool in resolving the diagnostic ambiguity often associated with these lesions and may be particularly effective in identifying spitzoid melanomas [55]. Their results agree with those of Googe et al., who reported in their study that only one of eleven Spitz nevi was diffusely positive, two were partially positive, and 73% of the Spitz nevi in their sample were PRAME-negative. Based on their experience, they suggested that diffuse PRAME reactivity in neoplastic melanocytes is typically indicative of malignancy [35]. In contrast, Raghavan et al. emphasised the need for caution in interpreting PRAME immunohistochemistry results in spitzoid neoplasms [54]. In their cohort of cases, the results showed that most Spitz nevi and atypical Spitz tumours completely lacked PRAME expression. However, it is important to note that some of these lesions (one Spitz nevus, one atypical Spitz tumour, and one spitzoid melanoma) occasionally showed diffuse PRAME expression. Consistent with the findings of Raghavan et al., Warbasse et al. conducted a study to evaluate the correlation between PRAME staining and FISH results in spitzoid and other difficult-to-diagnose melanocytic neoplasms. However, their conclusions were not as expected as they found that PRAME immunohistochemistry did not show a strong correlation with FISH results in spitzoid melanocytic neoplasms. Consequently, their study did not confirm the relevance of PRAME as an effective screening tool in this context. The researchers also highlighted the lack of consensus in the literature on the appropriate percentage of melanocytes with positive staining required to classify a lesion as ‘diffusely positive’. This ambiguity, they suggested, adds a level of complexity to the interpretation of PRAME test results in different studies [56]. Furthermore, Gerami et al. discovered a statistically significant correlation between PRAME expression and the diagnosis of atypical spitzoid melanocytic neoplasms. This implies that immunohistochemistry for PRAME can serve as a valuable supportive tool for suspected diagnoses. However, given occasional occurrences of false-positive and false-negative test results, it remains essential to correlate these findings with clinical and histologic observations, as well as results from additional tests, especially when interpreting diagnostically challenging spitzoid melanocytic neoplasms [57]. In McAfee’s 2023 study, an analysis of fifty-six cases of spitzoid neoplasms revealed that fifteen cases (27%) had diffuse PRAME expression. Of these, seven (47%) were FISH-positive, all of which were diagnosed as spitzoid melanoma. In contrast, the eight cases that showed positive PRAME expression but were FISH-negative included seven diagnoses of atypical Spitz tumours (AST) and one case was identified as a spitzoid melanoma, representing a discordant case. Importantly, this study did not find a statistically significant association between the PRAME expression and FISH status, as widespread PRAME expression was also found in several benign lesions [58]. The results of all these studies demonstrate the difficulties associated with relying on PRAME alone in the complex landscape of spitzoid lesions and highlight the importance of integrating multiple diagnostic approaches for a comprehensive evaluation.

## 6. PRAME Expression in Non-Melanoma Skin Cancer (NMSC)

Several studies have analysed the expression of PRAME in cutaneous non-melanoma carcinoma (NMSC) with interesting results. Elsensohn A et al. [26] analysed many histopathological entities in the NMSC spectrum, including well-to-poorly differentiated squamous cell carcinoma (SCC), basal cell carcinoma (BCC), basosquamous carcinoma, and Merkel cell carcinoma (MCC). Almost half of the samples showed some level of PRAME expression. Many cases showed low intensity, with staining observed in less than 25% of cells. BCCs were more likely to show staining, often accentuating the peripheral palisade cells [26]. Focusing on melanocytes of the dermal–epidermal junction (DEJ), 18% of NMSC samples showed a focal expression of high-intensity PRAME. SCCs were strongly associated with this pattern, with almost half (11/23, 48%) showing the random positivity of junctional melanocytes. The presence of rare, scattered melanocytes at the DEJ with high-intensity PRAME staining in 18% of NMSC samples examined, particularly in SCCs, suggests an association with extensive sun exposure. This finding has implications for the assessment of sample margins in chronically sun-damaged skin. Of note, BCC, SCC, and sebaceous carcinoma all showed low levels of PRAME immunoreactivity, with varying percentages of cases showing nuclear staining (BCC 59.4%, SCC 37.1%) [48]. PRAME expression in MCC, a rare and aggressive neuroendocrine neoplasm, merits discussion. In fact, MCC was found to be the second most common non-melanoma skin cancer to show some degree of PRAME expression (57% of lesions) and the most common NMSC tested showing staining in more than 25% of lesions. In fact, two Merkel cell carcinomas showed high-intensity staining in more than 75% of the tumour cells [26]. Melanoma and Merkel cell carcinoma both fall within the histopathological differential diagnosis of blue cell tumours, so it would be prudent to note that some Merkel cell carcinomas show a diffuse high-intensity expression of PRAME. Furthermore, PRAME expression in basal cell carcinoma and Merkel cell carcinoma is a potential pitfall that could lead to a misdiagnosis of malignant melanoma, especially in small biopsies and when melanoma is clinically suspected. Therefore, pathologists should be aware of the possible immunopositivity of PRAME in non-melanoma skin tumours [59]. The research suggests that PRAME expression in MCC may have potential diagnostic implications by helping to differentiate Merkel cell carcinoma from other cutaneous malignancies.

## 7. PRAME Expression in Sebaceous Carcinoma

PRAME appears to lack a specific informational value in the histopathological diagnostics of Sebaceous Carcinoma (SC), where other markers such as adipophylline can offer significant indications. However, in the context of subclassifying sebaceous carcinoma into grades I–II–III, following the guidelines of the latest WHO 2018, PRAME shows potential utility. It emphasises the presence of mature sebaceous differentiation foci, predominant in grades 1–2 and nearly absent in grade 3 of Sebaceous Carcinoma [60].

## 8. PRAME Expression in Soft Tissue Tumours

Regarding soft tissue tumours, a study is reported in the literature of 350 soft tissue tumours of >50 histotypes, in which PRAME immunoreactivity was graded from 0 to 4+ based on the percentage of positive cells. PRAME was expressed in 111 lesions, including various malignancies such as melanoma, synovial sarcoma, and myxoid liposarcoma, with varying degrees of diffuse positivity. Although the specificity of PRAME in soft tissue pathology is not perfect, it can serve as a valuable diagnostic adjunct in specific cases where expression patterns contrast with other lesions. Notably, several tumour types, including spindle cell lipoma and dermatofibrosarcoma protuberans, showed consistently negative PRAME expression. Furthermore, it could be useful in the differential diagnosis between melanoma and clear cell sarcoma (CCS) [59,61].

In addition, recent studies, including one investigating PRAME immunohistochemistry in dedifferentiated and undifferentiated melanoma, have strengthened the diagnostic value of PRAME. This study found that both primary and metastatic dedifferentiated and undifferentiated melanomas exhibited strong and diffuse nuclear PRAME staining, effectively distinguishing them from atypical fibroxanthoma and pleomorphic dermal sarcoma. The results underscore the role of PRAME as a first-line screening tool in the detection and management of these challenging melanoma cases and highlight its utility even in the absence of recognisable conventional melanoma precursors [62].

## 9. Implications for the Diagnosis and Prognosis of Melanoma

Melanoma-associated antigen PRAME, or Preferentially expressed Antigen in Melanoma, has emerged as a promising target in cancer research, particularly in the context of melanoma [1]. As we delve into the prospects of PRAME, it is evident that this antigen holds great potential in revolutionising melanoma diagnosis, prognosis, and treatment strategies. One of the significant prospects of PRAME lies in its diagnostic utility. As our understanding of the molecular landscape of melanoma advances, PRAME could serve as a valuable biomarker for early detection. The development of sensitive and specific assays to detect PRAME expression may be useful for selected diagnostic problems: the distinction of nevus from melanoma (in skin or lymph nodes) and the assessment of the margin clearance of melanomas, particularly in cases with a lentiginous in situ component such as seen in acral and lentigo maligna melanomas, enabling early intervention and improving patient outcomes [4]. It may be useful in the differential diagnosis between melanoma and some forms of sarcoma, such as clear cell sarcoma [44,61]. In addition to its diagnostic potential, PRAME is increasingly being recognised as a prognostic marker in melanoma [5]. Studies have indicated that elevated PRAME expression correlates with advanced disease stages and poorer survival outcomes. In fact, PRAME could help the diagnosis of MM also in a metastatic setting [6]. Incorporating a PRAME assessment into the current prognostic models may refine risk stratification, allowing clinicians to tailor treatment strategies based on individual patient profiles. This personalised approach to melanoma management could enhance the effectiveness of existing therapeutic modalities and facilitate the development of novel targeted therapies.

## 10. Therapeutic Perspectives

The therapeutic implications of PRAME in melanoma are particularly exciting. Retinoid-Based Therapy (RBT), even if a cheap and effective treatment option, now cannot achieve much clinical usage because of its variable responsiveness in the clinical outcomes. This clinical response variability may be attributed to the repression of retinoid receptors by the PRAME protein molecule. It is known that immunotherapy has revolutionised the landscape of cancer treatment, and PRAME is emerging as a promising immunotherapeutic target [10]. Strategies such as PRAME-specific T-cell-based immunotherapy [11] and PRAME-targeted vaccines [9] are being explored to harness the immune system (Table 3) and the ability to recognise and eliminate melanoma cells expressing PRAME. These approaches aim to induce a durable anti-tumour immune response, offering a more targeted and potentially less toxic alternative to conventional therapies [12]. Moreover, the combination of PRAME-targeted therapies with existing immunotherapeutic agents, such as immune checkpoint inhibitors, holds promise for synergistic effects. Combinatorial approaches may enhance the overall efficacy of immunotherapy in melanoma and overcome potential resistance mechanisms [13,14]. The evolving field of precision medicine in oncology is likely to benefit significantly from the integration of PRAME-targeted therapies, paving the way for more effective and individualised treatment strategies. As research on PRAME advances, it is crucial to address challenges such as antigen escape and immune tolerance [9]. Ongoing efforts to optimise the design of immunotherapeutic strategies and to understand the intricate interplay between the tumour microenvironment and PRAME expression are essential for translating the potential of PRAME into clinical success.

## 11. Conclusions (Table 4)

The comprehensive study of PRAME expression in various melanocytic and non-melanocytic lesions reveals its complex and nuanced role in diagnostic pathology. While benign mucosal melanocytic lesions and lymph node nevi show no PRAME expression, mucosal melanoma and lymph node metastases show increased PRAME expression. This differential expression pattern highlights the potential usefulness of PRAME in distinguishing benign from malignant melanocytic lesions. In other cases, such as Spitz tumours, the usefulness of PRAME as a diagnostic marker is unclear because PRAME is not consistently expressed in all Spitz nevi and atypical Spitz tumours and its absence does not necessarily indicate benignity. Furthermore, the role of PRAME in melanocytic nail lesions remains unclear as it has not been shown to be useful in differentiating benign from malignant lesions. In the context of acral lesions, PRAME has been shown to be a supportive marker, particularly in acral melanomas with histopathological uncertainty, offering higher sensitivity and specificity than p16. In uveal and conjunctival lesions, PRAME overexpression in uveal melanoma correlates with an increased risk of metastasis, while its role in conjunctival melanoma is less clear, but suggests potential as a biomarker. A critical consideration is non-melanoma skin cancer (NMSC), where PRAME expression varies, with basal cell carcinoma (BCC) showing a higher expression than squamous cell carcinoma (SCC). However, its expression in BCC and Merkel cell carcinoma (MCC) can lead to misdiagnosis, particularly in small biopsies or when malignant melanoma is suspected. In addition, the study, which included over 5800 tumour cases, characterised PRAME as a relatively unspecific marker, limiting its diagnostic utility in surgical pathology. In contrast, PRAME emerges as a critical marker for the diagnosis of undifferentiated melanoma, as highlighted by Hornick et al. This reinforces the importance of understanding the properties of PRAME and its application in histological diagnoses. Although PRAME positivity is a notable factor in the assessment of malignant melanoma, it cannot be directly equated with melanoma and requires a nuanced interpretation of its presence or absence in different lesions. Nevertheless, the detection of PRAME in malignant lesions offers significant value for potential immunotherapy targeting. In conclusion, PRAME is a testicular cancer antigen that serves as an important biomarker and potential therapeutic target in a variety of cancers. Its expression levels vary between different cancers, which may influence disease prognosis and treatment strategies. The future of the melanoma-associated antigen PRAME is promising and diverse. From its diagnostic potential to its role as a diagnostic and prognostic marker and as a target for innovative immunotherapies, PRAME is poised to play a pivotal role in the future of melanoma management. Future research and clinical trials will be essential to unlock the full therapeutic potential of PRAME and bring about a paradigm shift in the way we approach and treat melanoma.

**Table 4:** PRAME expression in skin cancers.

Type of Tumour	PRAME Expression	Reference
**Benign mucosal melanocytic lesions**	NO PRAME expression	[35,39]
**Mucosal melanoma**	↑ PRAME expression	[35,38,39]
**Lymph nodes nevi** **(nodal nevi)**	NO PRAME expression	[34]
**Lymph nodes metastases**	↑ PRAME expression	[34]
**Spitz nevi**	Googe et al. → PRAME expression is variable in Spitz nevi	[35]
	Raghavan et al. → NO PRAME expression in most of Spitz nevi and atypical Spitz tumours	[54]
	Warbasse et al. → PRAME is not a screening tool in Spitz nevi and Spitz tumours	[56]
**Nail melanocytic lesions**	PRAME is not useful in differentiating benign from malignant nail melanocytic lesions	[49,51,52]
**Acral lesions**	PRAME is a supportive marker in acral melanomas with histopathological uncertainties	[41]
	PRAME expression in acral melanomas has ↑ sen, ↑ spe than p16	[40]
	PRAME expression in acral lesions is: - positive in melanoma - negative in compounds, dysplastic, and Spitz nevi	[40]
**Uveal lesions**	↑ PRAME expression correlates with elevated risk of Uveal melanoma metastasis	[44]
**Conjunctival lesions**	PRAME is less understood, but can be a potential biomarker in conjunctival melanoma	[46]
**NMSC** - **BCC**	PRAME is expressed more in BCC than SCC	[26]
**NMSC** - **SCC**	PRAME in SCC is associated with extensive solar elastosis	[63]
**NMSC** - **MCC**	PRAME expression has a variable expression that can lead to erroneous diagnosis of MM in small biopsies or when MM is suspected (warning!)	[13]
**Soft Tissue Sarcoma**	PRAME could be useful in the differential diagnosis between melanoma and a subtype of Soft Tissue Sarcoma as the CCS	[61]
**Sebaceous Carcinoma**	PRAME does not support specific information in the histopathological diagnosis	[60]

## Figures and Tables

**Table 1:** PRAME: physiological expression in tissues.

	Reference
Testis	[4]
Ovary	[4]
Placenta	[4]
Adrenal gland	[4]
Endometrium	[4]

**Table 2:** PRAME: expression in cancer.

	Reference
Cutaneous melanoma	[7]
Non-melanoma skin cancer	[8]
Merkel cell carcinoma	[8]
Leukaemia; Acute Myeloid Leukaemia	[4]
Breast cancer	[2]
Ovarian cancer	[2]
Neuroblastoma	[5]
Non-small-cell lung cancer	[6]

**Table 3:** PRAME: therapeutic perspectives.

	Reference
T-cell-based immunotherapy	[8]
PRAME-targeted vaccines	[7]

## Abbreviations

**PRAME**	Preferentially expressed Antigen in Melanoma
**CT130**	Cancer Testis Antigen 130
**MAPE**	Melanoma Antigen Preferentially Expressed in tumours
**OIP-4**	Opa-interacting protein 4
**CTA**	Cancer/Testis Antigen
**AML**	Acute Myeloid Leukaemia
**LRR**	Leucine-Rich Repeats
**RA**	Retinoic Acid
**MITF**	Microphthalmia Transcription Factor
**NMSC**	Non-Melanoma Skin Cancer
**MCC**	Merkel Cell Carcinoma
**DEJ**	Dermal–Epidermal Junction
**SCC**	Squamous Cell Carcinoma
**BCC**	Basal Cell Carcinoma
**SUM**	Subungual Melanoma
**AM**	Acral Melanoma
**SMP**	Subungual Melanocytic Proliferation
**AST**	Atypical Spitz Tumour
**CCS**	Clear Cell Sarcoma
**RBT**	Retinoid-Based Therapy
**CAR-T**	Chimeric Antigen Receptor T cell
